# Targeted Therapy for Highly Desmoplastic and Immunosuppressive Tumor Microenvironment of Pancreatic Ductal Adenocarcinoma

**DOI:** 10.3390/cancers16081470

**Published:** 2024-04-11

**Authors:** Olamide T. Olaoba, Ming Yang, Temitope I. Adelusi, Tessa Maidens, Eric T. Kimchi, Kevin F. Staveley-O’Carroll, Guangfu Li

**Affiliations:** 1 Department of Surgery, University of Missouri, Columbia, MO 65212, USA; otowbx@missouri.edu (O.T.O.); yangmin@umsystem.edu (M.Y.); tah6x@umsystem.edu (T.I.A.); tdmnm2@umsystem.edu (T.M.); kimchie@health.missouri.edu (E.T.K.); 2 Department of Molecular Microbiology and Immunology, University of Missouri, Columbia, MO 65212, USA; 3 Roy Blunt NextGen Precision Health Institute, University of Missouri, Columbia, MO 65212, USA; 4 Harry S. Truman Memorial Veterans’ Hospital, Columbia, MO 65201, USA; 5 Ellis Fischel Cancer Center, University of Missouri, Columbia, MO 65212, USA

**Keywords:** PDAC, targeted therapy, desmoplasia, immunotherapy, tumor microenvironment

## Abstract

**Simple Summary:**

Pancreatic ductal adenocarcinoma (PDAC) is characterized by a highly desmoplastic and extreme immunosuppressive tumor microenvironment (TME), which underlines the chemoresistant mechanism in PDAC. The TME components contain actionable targets for drug development. The aim of this review is to discuss the complexity of the TME, highlighting actionable drug targets that have been identified and targeted in preclinical studies and clinical trials.

**Abstract:**

Pancreatic ductal adenocarcinoma (PDAC) is a highly lethal malignancy with a very poor prognosis. Despite advancements in treatment strategies, PDAC remains recalcitrant to therapies because patients are often diagnosed at an advanced stage. The advanced stage of PDAC is characterized by metastasis, which typically renders it unresectable by surgery or untreatable by chemotherapy. The tumor microenvironment (TME) of PDAC comprises highly proliferative myofibroblast-like cells and hosts the intense deposition of a extracellular matrix component that forms dense fibrous connective tissue, a process called the desmoplastic reaction. In desmoplastic TMEs, the incessant aberration of signaling pathways contributes to immunosuppression by suppressing antitumor immunity. This feature offers a protective barrier that impedes the targeted delivery of drugs. In addition, the efficacy of immunotherapy is compromised because of the immune cold TME of PDAC. Targeted therapy approaches towards stromal and immunosuppressive TMEs are challenging. In this review, we discuss cellular and non-cellular TME components that contain actionable targets for drug development. We also highlight findings from preclinical studies and provide updates about the efficacies of new investigational drugs in clinical trials.

## 1. Introduction

Pancreatic ductal adenocarcinoma (PDAC) is a lethal malignancy with a dismal prognosis. Generally, pancreatic cancer is the sixth leading cause of cancer-related death globally and the third leading cause of cancer-related mortality in the United States [1]. PDAC patients have a very rare chance of early diagnosis, and therefore, only a few present with low-stage tumors. Surgery, which is one of the common treatment approaches for resectable tumors, is beneficial to only a small percentage of PDAC patients and provides a median survival of 17 to 23 months [2]. Further, the use of chemotherapy has shown promising efficacy in different types of cancer. However, PDAC is highly resistant to most treatment options, including chemotherapy.

One of the reasons for failed therapeutic intervention in PDAC is the prominence of a stromal microenvironment with striking cellular and material heterogeneity, which constitutes a mechanical barrier that impedes successful drug delivery [3]. Consequent to desmoplasia, immunomodulatory cells and stromal support cells are incessantly challenged by the aberrant signaling of pancreatic cancer cells, leading to a hypoxic tumor microenvironment (TME), ECM deposition, the suppression of antitumor immunity, and the advancement of tumorigenesis [4]. These counterbalances in immune and cellular architectures underline the highly resistant TME of PDAC. Most therapeutic approaches that aim to deconvolute the desmoplastic stroma and short-circuiting immunosuppressive pathways have not been successful [5]. Therefore, in this review, we discuss the structural composition, physiology, and pathways that reinforce the tumor microenvironment of PDAC. We also highlight TME components that have been targeted in preclinical studies and provide updates about the efficacies of new investigational drugs in clinical trials.

## 2. Highly Desmoplastic Tumor Microenvironment in PDAC

### 2.1. Targeting the ECM Components

**Figure 1:**
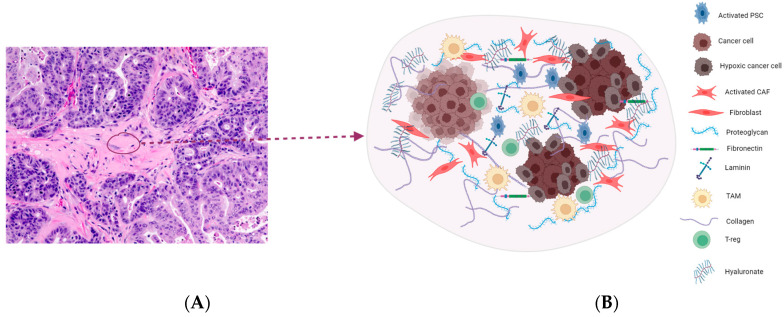
(**A**) (20×) H&E staining of a patient-derived xenograft model of PDAC imaged in our laboratory. The tissue section shows an area of the desmoplastic reaction which is characterized by a dense aplastic appearance. Pancreatic stellate cells, fibroblasts, and suppressor cell infiltrates colonize this area with an extensive deposition of extracellular matrix components, leading to increased interstitial fluid pressure and reduced perfusion (**B**). Overall, this feature prevents drug access into the TME, thereby limiting therapeutic efficacy in pancreatic cancer.

One of the pathological features of pancreatic cancer is desmoplasia, coined from two Greek words, *desmo*, meaning ‘band’ or ‘fastening’, and *plassein*, meaning to ‘form’ or ‘mold’. The desmoplastic reaction is characterized by an extensive proliferation of myofibroblast-like cells and the deposition of extracellular matrix components [6]. These dramatic events lead to the formation of dense fibrous connective tissues that comprise cellular and non-cellular components. The cellular components include alpha smooth muscle actin-positive fibroblasts, activated pancreatic stellate cells (PSCs), and immune cell infiltrates. On the other hand, extracellular matrix (ECM) proteins like collagen, laminin, fibronectin, integrin, and proteoglycan hyaluronan represent the non-cell components of the desmoplastic reaction (Figure 1). These components contribute to increased tumor heterogeneity, reduced plasticity, and elevated interstitial fluid pressure. Overall, the fibrous tissue formed creates a mechanical barrier that is fundamental to the development of resistance to chemotherapy in pancreatic cancer patients [6,7]. Therefore, targeting the desmoplastic reaction components represents an efficient therapeutic intervention in pancreatic cancer.

### 2.2. Hyaluronic Acid

Hyaluronic acid (HA) is a linear glycosaminoglycan that is composed of a repeating disaccharide unit of glucuronic acid and *N*-acetylglucosamine. It is synthesized by HA synthetases and degraded by hyaluronidases. The balance between the synthesis and degradation of HA is an important factor in health and diseases. As an ECM component, high concentrations of HA have been linked to disease progression. In human PDAC tissue, there is a 12-fold increase in the amount of HA secreted compared to normal pancreases [8]. The expression of HA has been shown in both PDAC stroma and tumor cells. HA refuels the hexosamine pathway [9], this pathway is very active in PDAC. Specifically, HA enhances the proliferation, survival, and growth of pancreatic tumors. It is the major contributor of interstitial fluid pressure that decreases tumor perfusion, hence leading to limited access to chemotherapy. Thus, targeting HA may provide clinical benefits to pancreatic cancer patients.

Several approaches have been used to target HA in different studies, including targeting the inhibition of HA synthesis, antagonizing HA signaling, and depleting HA in the stroma. 4-methylumbelliferone (4-MU), also called hymecromone, has been reported to inhibit HA synthesis in many preclinical models. 4-MU treatment hampered the cell migration of a PDAC cell line [10] and enhanced anticancer efficacy of chemotherapy by reducing HA in a stroma-rich ECM [11]. In clinical trials, the efficacy of 4-MU has been evaluated in bile duct obstruction diseases and COVID-19 patients but not pancreatic cancer patients. Currently, there are no clinically approved drugs or ongoing clinical trials that specifically target HA synthesis in pancreatic cancer patients. Thus, further preclinical and clinical studies are required to identify and evaluate targeted inhibitors of HA synthesis in pancreatic cancer patients.

**Figure 2:**
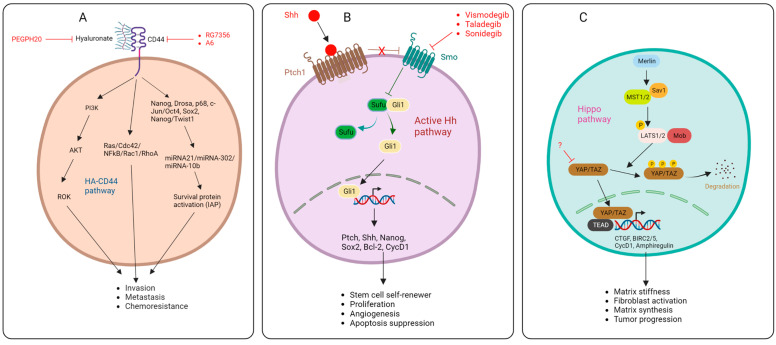
HA-CD44 pathways (**A**), Sonic Hedgehog pathway (**B**), Hippo pathway (**C**), and targets of inhibitors.

Targeting the HA signaling pathways is one of the therapeutic approaches for targeting HA (Figure 2). This strategy prevents the effect of HA signaling. Cell surface receptors such as CD44 and CD168 can be docked by HA and regulate PDAC invasion and metastasis [12]. HA receptors are highly immunogenic. Monoclonal antibodies that can target these receptors have been developed in several clinical trials. In a phase 1 dose escalation study, RG7356, a recombinant anti-CD44 humanized monoclonal antibody was administered in escalating doses to 65 patients with high CD44-expressing solid tumors. While modest clinical efficacy was achieved in 21% of patients (13/61 patients achieved stable disease), there were records of dose-limiting toxicities, which include headache and febrile neutropenia [13]. In several recurrent carcinomas, the administration of the CD44-targeting agent A6—an urokinase-derived peptide—yielded minimal activity [14], whereas a phase 1 clinical trial of bivatuzumab mertansine in patients with incurable squamous cell carcinoma of the head and neck or esophagus was terminated. This is because clinical development was discontinued before the maximum tolerable dose (MTD) was reached [15]. However, the safety and clinical efficacy of small molecule inhibitors of CD44 have not been investigated in clinical trials. On the other hand, the receptor for hyaluronic acid-mediated motility (RHAMM), also known as CD168, has been targeted in clinical trials (Table 1). RHAMM plays a vital role in growth, differentiation, and motility. It is one of the leukemia-associated antigens (LAAs) that are highly expressed in patients with myeloid leukemia [16]. The expression of RHAMM has also been correlated with genetic instability in patients with multiple myeloma and lymphocytic leukemia [17,18]. Importantly, the preferred target of immunotherapy is RHAMM due to its antigenicity. Many clinical studies have also used RHAMM-derived peptides to vaccinate cancer patients [16,17]. This approach has increased the efficacy of immunotherapy. Thus, RHAMM-derived vaccination may be a potent therapeutic strategy to increase stroma perfusion and reduce chemoresistance in pancreatic cancer.

Previously in this section, we explained the critical role of HA accumulation in interstitial fluid pressure elevation, which reinforces the stromal mechanical barrier, consequentially impairing efficient drug delivery to tumors. This chemoresistance phenomenon could be abolished by depleting HA in the stroma. In a mouse model, the intravenous infusion of PEGPH20 was shown to deplete stromal HA, normalized interstitial pressure, and improved the efficacy of gemcitabine [19]. PEGPH20 is a Pegylated recombinant humanized hyaluronidase which has been investigated in several phase 1 and II trials [20,21,22,23]. In 2023, in multiple open-label, randomized, phase Ib/II (MORPHEUS) trials (NCT03193190, NCT03281369), the early efficacy and safety profile of atezolizumab plus PEGPH20 was investigated in comparison with chemotherapy in PDAC patients. The objective response rate (ORR) of atezolizumab + PEGPH20 was 6.1% (95% CI, 1.68–14.8%), compared to the ORR of 2.4% (95% CI, 0.06–12.57%) in chemotherapy-treated arm. However, the combination of atezolizumab and PEGPH20 offered limited clinical benefits to PDAC patients. In fact, more than 61% of the patients developed grade 3/4 adverse events [24]. Due to some successes in preclinical models and early-phase clinical trials, the efficacy of PEGPH20 has been evaluated at phase III in HA-high stage IV pancreatic cancer patients. PEGPH20 increased tumor perfusion by decreasing interstitial tumor pressure and increasing tumor plasticity [25]. In another phase III trial, the addition of PEGPH20 to nab-paclitaxel/gemcitabine in HA-high metastatic PDAC patients increased ORR but not PFS or OS [26]. Apparently, PEGPH20 has been investigated in several trials and in pancreatic cancer settings; this suggests that PEGPH20 may be combined with several neoadjuvant therapies to increase their potencies in pancreatic cancer patients. However, more clinical studies are required to validate its efficacy.

### 2.3. Integrins

Integrins are cellular adhesion receptors that play vital roles in cell-to-cell and cell–extracellular matrix interactions. Integrins are key components of cell migration systems. Studies have shown that integrins play roles in the anchorage-independent survival of circulating tumor cells. Specifically, integrins mediate the stiffness, sensing, and remodeling of stroma, thereby promoting invasion and resistance to therapy [27]. Although targeting integrins is a complex therapeutic approach, emerging strategies are focusing on personalized approaches that target specific integrin subunits (Table 1). CEND-1 is a cyclic peptide that targets αV integrins and neurophilin-1. The co-administration of CEND-1 and chemotherapy has been shown to enhance tumor delivery. In an open-label, multicenter, phase 1 study, the safety, tolerability, and efficacy of CEND-1 in combination with nab-paclitaxel and gemcitabine were evaluated in PDAC patients with histologically confirmed metastasis (NCT03517176). The combination of CEND-1 and nab-paclitaxel/gemcitabine was well tolerated, with there being no CEND-1 dose-limiting toxicities in the safety population. Most grade 3/4 adverse events include neutropenia (55%), anemia (26%), leukopenia (16%), and pulmonary embolism (13%). In the efficacy arm, 59% of the patients achieved objective response, including 1 complete response and 16 partial responses [28]. In another study, the safety and efficacy of E7820 was investigated in preclinical and clinical settings. E7820 is an orally active inhibitor of α2 integrin. Mechanistically, E7820 prevents the mRNA expression of α2 integrin. In mice bearing KP-1 tumors, E7820 moderately inhibited α2 integrin expression, and this was correlated with tumor stasis. This was also recapitulated in a phase 1 study; the administration of 100 mg qd (MTD) E7820 strongly inhibited α2 expression [29].

### 2.4. Sonic Hedgehog Pathway

One of the signals that maintains the active state of pancreatic tumor stroma is the developmental protein Sonic Hedgehog protein (SHH). Tumor cells upregulate SHH, leading to the activation of the surrounding stroma and tumor progression. In one particular study, SHH-dependent factors were identified through survival and immunohistochemistry analyses of non-microdissected tissue from PDAC patients. These factors include CDA, EDIL3, ITGB4, PLAUR, and SPOCK1 [30]. Targeting one of these factors may represent an efficacious strategy for abolishing the downstream effect of the SHH signal in pancreatic stroma. The activation of the Hedgehog pathway (Hh) requires three proteins, namely the Hh ligand, Patched (Ptch), and Smoothened (Smo). Without the Hh ligand, Ptch1 is found at the base of the primary cilia, where it represses the activities of Smo. Ptch1’s inhibitory activities on Smo prevent it from translocating to the primary cilia, leading to the proteolytic cleavage of glioma-associated oncogene (GLiFL) to GLi repressor GLiR. Protein kinase A (PKA), glycogen synthase kinase 3 (GSK3), and casein kinase 1 (CK1) phosphorylates GliR, this allows GliR to transcriptionally repress Hh target genes. However, when the Hh ligand is present, it binds to the Ptch1 protein. The Hh–Ptch1 complex is then internalized and degraded by lysosomal enzymes. This relieves Smo and allows it to transmit downstream signals via the cytoplasmic protein complex comprising kinesin protein (Kif7), suppressor of fused (Sufu), and GliFL, leading to the release of GLi activator (GLiA) and nuclear translocation to activate Hh target genes (Figure 2) [31].

Since the Hh pathway is largely upregulated in pancreatic adenocarcinoma stem cells, targeting this pathway may offer therapeutic benefits to pancreatic cancer patients. Many clinical studies based on targeting the Hh pathway have yielded promising results in pancreatic cancer and other cancer types (Table 1) [32,33,34]. Vismodegib (GDC-0449) is an orally administered small molecule inhibitor of the Hh pathway. In a pilot clinical study that involved 25 enrolled PDAC patients, pretreatment biopsy showed SHH expression in 75% of the patients. However, post-treatment, GL1 and Ptch1 decreased in 95.6% and 82.6% of 23 patients. Fibrosis (45.4% of 22) and Ki-67 (52.9% of 17) also decreased. There was a significant correlation between CSCs, fibrosis, SHH, Ki-67, GL1, Ptch1, and survival [35]. Following its promising efficacy, vismodegib was also evaluated in a phase I/II trial as a monotherapy and part of a combination therapy [36]. In another trial, escalating doses of taladegib, an oral inhibitor of Smoothened, led to the high-level inhibition of Gli1 transcript after 15 and 30 days [37]. Besides pancreatic cancer, inhibitors of the Hh pathway have also shown promising outcomes. A phase II trial evaluated the Smo inhibitor sonidegib in medulloblastoma patients; 50% of patients with an initially activated Hh pathway achieved complete or partial remission [38]. Sonidegib and other inhibitors that have been evaluated in other cancer patients may be repurposed and evaluated in the setting of PDAC.

### 2.5. Hippo Pathway

Another signal that regulates fibroblast activation are the transcriptional effectors of the Hippo pathway, the Yes-associated protein (YAP), and the transcriptional activator with a PDZ-binding motif (TAZ). YAP-TAZ transduces the major cues of the Hippo shunt, leading to the regulation of matrix stiffness, fibroblast activation, and matrix synthesis. The expression and accumulation of TAZ has been shown in the nuclei of spindle-like fibroblasts. In in vitro studies, the depletion of YAP and TAZ abolished fibroblast function and reduced matrix synthesis [39]. The YAP-TAZ pathway (Hippo pathway) was shown to modulate the phenotype of pancreatic stellate cells and promote the recruitment of tumor-associated macrophages and myeloid-derived suppressor cells. The importance of the Hippo pathway in pancreatic cancer development was demonstrated when YAP-TAZ cues promoted tumor progression despite the absence of KRAS mutation. This shows that the Hippo pathway is critical to tumor growth in pancreatic cancer. The Hippo pathway is characterized by sequences of molecular cascades that activate the Hippo transducers YAP-TAZ (Figure 2). YAP-TAZ accumulates in the nucleus and interacts with TEA transcriptional factors TEADs, leading to the induction of gene expression. In pancreatic cancer, the outcome of this pathway leads to tumorigenesis, tumor growth, EMT, stroma generation, immunomodulation, and chemoresistance [40]. Therefore, inhibitors of Hippo transducers may represent a breakthrough in breaking the stroma barrier and abolishing drug resistance. Unfortunately, there are currently no advances in this area. In a preclinical study, Higashi and colleagues showed that TAZ expression was significantly downregulated in HFL cells after treatment with Fluvastatin and simvastatin [41]. Future studies should focus on the development of more inhibitors of this pathway to overcome the stroma barrier.

### 2.6. Cancer-Associated Fibroblasts (CAFs)

CAFs, previously identified as a prominent component of desmoplasia, have recently emerged as a therapeutic target in PDAC [42]. The role of CAFs in immune evasion and therapeutic resistance has made them a desirable target; however, effective clinical translation of this research has yet to show definitive success. The primary challenges of CAF-targeting treatments appear to result from the heterogenicity and complex functions of CAFs in the TME and a lack of complete mechanistic understanding [43]. Early research focused on the complete eradication/inhibition of CAFs in the TME, with ineffective and detrimental results in the hosts. Initially, targeted therapies included the ablation of α-SMA+ and anti-FAP therapies [44,45]. A study from early 2014 utilized transgenic mice to delete α-SMA+ myofibroblasts in pancreatic cancer, resulting in diminished animal survival correlated with increased CD4+Foxp3+ Tregs [44]. An early 2013 study exhibited the detrimental effects of non-specific anti-FAP therapy in two different mouse strains due to the FAP-reactive T cells present in bone marrow mesenchymal stem cells [45]. Current research has since shifted to increasing the specificity of therapies to target only CAFs and targeting subpopulations of CAFs to inhibit protumorigenic properties while maintaining tumor-suppressive properties [46].

It has been well established in the literature that STAT3 activation in iCAFs plays a critical role in the poor prognosis of cancer, making it a desirable target for treatment [47]. Ruxolitinib, a JAK inhibitor, failed in phase III clinical studies after it failed to show improved survival rates in patients with pancreatic cancer when combined with capecitabine [48]. Other approaches to targeting STAT3 activation were deemed necessary; these included targeting cytokine receptors upstream. Clinical trials of Anakinra, an IL-1R antagonist, are currently recruiting for phase II trials (NCT04926467) after showing initial success in combination with gemcitabine in human cell lines and nude mice models [49]. High expression levels of circFARP1 have been observed in advanced PDAC patient tumors and identified as playing a role in STAT3 activation, which increases GEM resistance [50]. This may be a desirable therapeutic target in the CAF STAT3 combination to enhance the effectiveness of GEM treatment in PDAC.

CAFs secrete FGF, which supports tumor growth and progression, and FGFR1–4 expression has been reported in pancreatic cancer, making them promising new therapeutic targets [51]. There are currently few trials for PDAC-targeting FGF/FGFR1–4, but there have been encouraging results for FGFR inhibitors in early clinical trials of different cancers. In a phase I trial, Futibatinib demonstrated the clinical efficacy and tolerability of FGFR1–4 inhibitors in many different tumors [52]. After demonstrating clinical efficacy across tumors, including pancreatic cancer, Pemigatinib has moved into phase II and III trials [53]. A phase Ib trial of Dovitinib reported clinical activity with gemcitabine and capecitabine in pancreatic patients, but no phase II trials are currently ongoing [54]. Lenvatinib reportedly showed antitumor activity in patients with biliary tract cancer in a phase II trial, so this FGFR1–4 inhibitor may have potential in PDAC treatment as well [55]. There are multiple ongoing phase I and II studies of Futibatinib, including one featuring patients with advanced KRAS mutant cancer. The presence of FGF/FGFR1–4 in pancreatic cancers may also be a useful therapeutic approach for PDAC.

CAF-mediated CXCL12/CXCR4 is targeted because of its established role in invasion and metastasis in several types of cancer [56]. Plerixafor (AMD3100), a CXCR4 inhibitor, yielded positive results in a phase II trial on patients with metastatic pancreatic cancer [57]. However, these results were not indicative of clinical efficacy because the inhibition of CXCR4 was limited to only one week, so clinical trials with long-term inhibition are still necessary to determine its therapeutic potential. Motixafortide (BL-8040) combined with pembrolizumab in a phase II COMBAT trial enhanced the efficacy of chemotherapy in patients with pancreatic cancer, and another phase II trial for patients with PDAC (NCT04543071) is currently recruiting [58]. Targeting CAFs’ ability to remodel the ECM is another therapeutic approach currently being used. Losartan has been reported to inhibit collagen I synthesis and enhance nanoparticle drug efficacy in human pancreatic tumor models in mice [59]. The FDA has not approved losartan for pancreatic cancer treatment, but an investigational phase II clinical trial for losartan combined with FLOFIRINOX and 9-Ing-41 is currently recruiting to test the potential benefits in PDAC (NCT05077800).

Previous studies in the literature have reported high levels of SMO expression in CAFs, indicating that the SHH pathway is activated, making the paracrine mechanism a target [60]. In a phase I clinical trial for combination therapy using SMOi sonidegib and docetaxel chemotherapy to treat triple-negative breast cancer, 3 of 12 patients derived clinical benefit, and 1 of 12 experienced complete response [61]. There are not currently any phase II trials ongoing in relation to these findings. This was a small cohort, and phase I trials do not determine therapeutic efficacy, but this therapy has the potential to be applied to PDAC treatment. ProAgio, a protein that targets integrin α_v_β_3_-expressing cells and induces apoptosis, has been used for CAF depletion, resulting in the enhancement of chemotherapy agents used in combination in triple-negative breast cancer murine models [62].

A study of WNT2 in both OSCC and CRC allograft tumors found that anti-WNT2, which suppresses antitumor T-cell response in DC via the SOCS3/p-JAK2/p-STAT3 signaling cascades, enhances the therapeutic response in anti-PD1 therapy [63]. This finding warrants the further exploration of its clinical application, and a phase I and II trial of ST316 is currently recruiting (NCT05848739).

Following the initial success of clinical trials of CART-cell therapies in patients with multiple myeloma, it was observed in phase II trials that patients would experience poor remission and cytotoxic effects with its application [64]. A study using multiple myeloma models found preclinical success when using a combination of CART therapy and targeting BM-CAFs with both BCMA and SLAMF7 [65]. However, this study recommended continuing clinical trials with caution because of potential toxicities and the possibility of BM-CAFs being dirty targets. Overall, the heterogeneity of CAFs increases the difficulty in the application of specific therapeutic targets across different cancers, but effective targets may still guide use against PDAC.

**Table 1:** Inhibitors of the stromal barrier.

Target	Agent(s)	Phase	Disease(s)	Mechanism	Reference(s)
Hyaluronic Acid (HA)	Hymecromone (4-MU)		Bile duct obstruction and COVID-19	Inhibit HA synthesis and impede migration	[10,11]
Hyaluronic Acid (HA)	RG7356	I	High CD44-expressing solid tumors	Selective binding on CD44 inhibits growth	[13]
Hyaluronic Acid (HA)	A6	II	Recurrent EOC/FTC/PPC	Binds to CD44, reducing CD44 bonding and activity	[14]
Hyaluronic Acid (HA)	Bivatuzumab + Mertansine	I	Squamous cell carcinoma of head, neck, or esophagus	Binding to CD44v6 to enable intracellular release and induce miotic arrest and cell death	[15]
Hyaluronic Acid (HA)	RHAMM peptide (CD168)	I/II	Chronic lymphocytic leukemia	Hinders miosis	[17,18]
Hyaluronic Acid (HA)	PEGH20	I/II		Degrades HA in the stroma	[20,21,22,23]
Hyaluronic Acid (HA)	PEGH20 + atezolizumab	Ib/II	PDAC	Degrades HA in the stroma	NCT03193190 NCT03281369
Hyaluronic Acid (HA)	PEGH20	III	HA-high stage IV pancreatic cancer	Increases tumor profusion by decreasing tumor pressure and increasing tumor plasticity	[25]
Hyaluronic Acid (HA)	PEGH20 + Nab-paclitaxel/gemcitabine	III	HA-high metastatic PDAC	Degrades HA in the stroma	[26]
αV Integrins and neurophilin-1	CEND-1 + Nab-paclitaxel/gemcitabine	I	PDAC	Interacts with αV integrins and activates drug transport via neurophilin-1	NCT03517176
α2 Integrins	E7820	I	Malignant solid tumors or lymphomas	Prevents mRNA expression of α2 integrins	[29]
SHH	Vismodegib (GDC-0449)	I/II	PDAC and medulloblastoma	Inhibits SMO to inhibit Hh signaling pathway	[35,36]
SHH	Taladegib	I	Advanced solid tumors	Inhibition of Hh signaling pathway mediated by protein SMO	[37]
SHH	Sonidegib (LDE225)	II	Medulloblastoma	Inhibitor of Smoothened, preventing downstream activation	[38]
Cancer-associated fibroblasts	Ruxolitinib + capecitabine	III	Pancreatic cancer	Inhibition of JAK1 and JAK2, impeding cell signaling	[66]
Cancer-associated fibroblasts	Anakinra	II	PDAC	IL-1R antagonist	NCT04926467
Cancer-associated fibroblasts	Futibatinib	I/II	*FGFR*-aberrant tumors	Anti-FGFR inhibits FGFR signaling pathways	[52]
Cancer-associated fibroblasts	Pemigatinib	II/III	Refractory advanced malignancies	Anti-FGFR inhibits FGFR signaling pathways	[53]
Cancer-associated fibroblasts	Dovitinib + gemcitabine and capecitabine	Ib	Pancreatic cancer	Anti-FGFR inhibits FGFR signaling pathways	[54]
Cancer-associated fibroblasts	Lenvatinib	II	Biliary tract cancer	Anti-FGFR inhibits FGFR signaling pathways	[55]
Cancer-associated fibroblasts	Plerixafor (AMD3100)	II	Metastatic pancreatic cancer	CXCR4 inhibition, activating intratumoral immunity	[57]
Cancer-associated fibroblasts	Motixafortide (BL-8040) + pembrolizumab	II COMBAT trial	Pancreatic cancer	Inhibition of CXCR4 activation, increasing intratumoral immunity	[58] NCT04543071
Cancer-associated fibroblasts	Losartan + FLOFIRNOX and 9-Ing-41	II	PDAC	Inhibit collagen I synthesis	NCT05077800
Cancer-associated fibroblasts	SMOi sonidegib + docetaxel	I	Triple-negative breast cancer	Inhibition of signaling and inhibition of microtubule assembly	[61]
Cancer-associated fibroblasts	ProAgio	Preclinical	Triple-negative breast cancer	CAF depletion by targeting integrin αvβ3-expressing cells and inducing apoptosis	[62]
Cancer-associated fibroblasts	ST316	I/II	Advanced solid tumors	Suppress transcription of Wnt genes	NCT05848739
Cancer-associated fibroblasts	Dual-targeting CART-cells	Preclinical	Multiple myeloma	CART signaling, targeting malignant plasma cells and CAFs	[65]

## 3. Profound Immunosuppressive TME and Immunotherapy in PDAC

**Figure 3:**
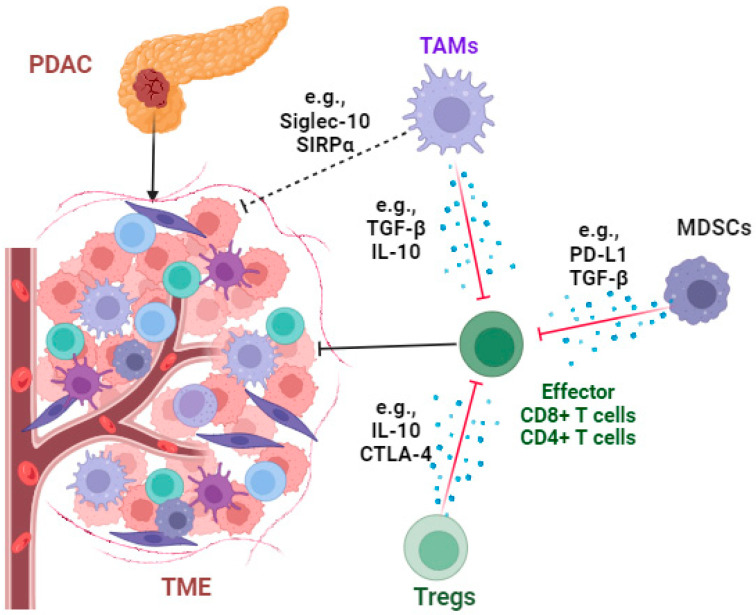
The immunosuppressive tumor microenvironment of PDAC. The infiltration of immunosuppressive cells such as tumor-associated macrophages (TAMs), regulatory T cells (Tregs), and myeloid-derived suppressor cells (MDSCs); the secretion of immunosuppressive cytokines such as transforming growth factor β (TGF-β) and interleukin 10 (IL-10); the expression of immune checkpoints such as programmed death-ligand 1 (PD-L1) and cytotoxic T-lymphocyte-associated protein 4 (CTLA-4); and “don’t eat me” signaling molecules such as signal-regulatory proteins (SIRPα) and sialic acid-binding Ig-like lectin 10 (Siglec-10), together, play vital roles in immune evasion in the PDAC microenvironment. The diagram of this figure was created using Biorender (https://www.biorender.com/ (accessed on 15 September 2023)).

The infiltration of immunosuppressive cells (Figure 3) such as tumor-associated macrophages (TAMs), regulatory T cells (Tregs), and myeloid-derived suppressor cells (MDSCs); the secretion of immunosuppressive cytokines such as transforming growth factor β (TGF-β) and interleukin 10 (IL-10); and the expression of immune checkpoints such as programmed death-ligand 1 (PD-L1) and cytotoxic T-lymphocyte-associated protein 4 (CTLA-4) play vital roles in immune evasion in PDAC [67,68]. By targeting these cells and molecular signaling pathways, the survival of PDAC patients can be improved [69]. However, mono-immunotherapy fails to achieve the promising clinical benefit in patients with PDAC due to the highly immunosuppressive tumor microenvironment (TME) [70]. Here, we discuss various targeted therapies on cellular and molecular levels and evaluate their outcomes.

### 3.1. Targeting Immunosuppressive Cells

#### 3.1.1. Tumor-Associated Macrophages (TAMs)

TAMs are one of the most abundant immune cells in the TME of PDAC. These TAMs are M2-like macrophages and produce immunosuppressive cytokines such as IL-10 and TGF-β to inhibit cytotoxic T-cell functions and induce pancreatic fibrosis by enhancing the production of ECM proteins [71,72,73]. TGF-β1 secreted from TAMs plays a key factor in inducing PD-L1 expression in PDACs by enhancing the interaction between pyruvate kinase M2 (PKM2) and the signal transducer and activator of transcription 1 (STAT1) [74]. The phagocytosis function of TAMs is significantly compromised by “don’t eat me” signaling, such as the axis of signal-regulatory proteins (SIRPα) on TAMs–cluster of differentiation 47 (CD47) on PDAC cells [75,76]. Similarly, PDAC cells (e.g., Panc1 cells) express CD24 to escape macrophage phagocytosis by binding with the inhibitory receptor sialic acid-binding Ig-like lectin 10 (Siglec-10), expressed by TAMs [77]. In addition, TAMs can promote tumor cell metastasis and induce therapeutic resistance. For example, NLR family pyrin domain containing 3 (NLRP3) activation induced the M2-like macrophage polarization of TAMs in a murine model of PDAC to promote cancer cell lung metastasis [78]. Therefore, targeting TAMs and their associated factors can improve PDAC therapy.

CD40 is a TNF receptor superfamily member expressed by immune and non-immune cells [79]. In human macrophages, CD40 expression is induced through the IL-6-mediated upregulation of STAT3 and HIF, whereas in mice, STAT-1α and ETS play roles in the induction of CD40 expression [80]. CD40 on antigen-presenting cells, like DC, can bind CD40L, inducing its activation and the subsequent priming of cytotoxic T lymphocytes, leading to an enhanced antitumor response [81]. In fact, CD40 knockout mice showed impaired T cell priming and exhibited a markedly higher occurrence of spontaneous tumors [82]. Thus, agents that can activate CD40 may be beneficial to pancreatic cancer patients. Several studies have demonstrated the ability of CD40 activation to modulate the TME [83]. Specifically, CD40 has been shown to improve the efficacy of checkpoint inhibitors in pancreatic cancer patients [84]. Due to the successes of preclinical studies, CD40 agonists like selicrelumab have been evaluated in clinical trials. In a particular neoadjuvant clinical trial, the administration of neoadjuvant selicrelumab enhanced T-cell proliferation, reduced M2-like TAMs and fibrosis, and increased the systemic frequency of CXCL10 and CCL22 compared to treatment-naive PDAC patients or patients administered with other neoadjuvant [85].

Other TAM-targeting therapies have been developed. A phase 1b trial (Clinicaltrials.gov, NCT01413022) blocked the recruitment of TAMs into the TME of PDAC using CCR2 inhibitor PF-04136309 in combination with chemotherapy FOLFIRINOX (oxaliplatin and irinotecan plus leucovorin and fluorouracil), achieving an objective tumor response in 49% (16) of 33 patients, with local tumor control in 97% (32) of 33 patients, showing better treatment response compared to the FOLFIRINOX-treated group [86]. Another clinical trial (NCT02345408) showed that treatment with a CCR2-specific antagonist, CCX872-B, plus FOLFIRINOX resulted in an overall survival (OS) rate of 29% at 18 months by decreasing peripheral blood monocytes without causing safety issues [87]. The depletion of TAMs is also an option to improve the antitumor efficacy of immunotherapies. For example, a phase 1 clinical trial (NCT02777710) evaluated the safety and activity of a tyrosine kinase inhibitor of colony-stimulating factor 1 receptor (CSF1R) (pexidartinib) in combination with anti-PD-L1 antibody (durvalumab) in patients with metastatic/advanced pancreatic or colorectal cancers, and no unexpected events were shown in the group who received the combined therapy [88]. Pexidartinib is applied to deplete M2 macrophages in the TME of PDAC. A clinical trial (NCT05558982) is recruiting to evaluate the efficacy of macrophage activator BXCL701 in combination with pembrolizumab in patients with PDAC.

#### 3.1.2. Regulatory T Cells (Tregs)

Tregs are highly infiltrated in PDAC, contributing to the immunosuppressive TME and causing resistance to immunotherapy by secreting immunosuppressive cytokines (e.g., TGF-β) and expressing immune checkpoints (e.g., programmed cell death protein 1 [PD-1], CTLA-4, T cell immunoglobulin and mucin domain-containing protein 3 [Tim-3], and lymphocyte activation gene-3 [LAG-3]) [68,89]. Ipilimumab, an anti-CTLA-4 monoclonal antibody, has been shown to increase antitumor immunity by inhibiting Tregs due to their constitutive expression of CTLA-4 [90]. Unfortunately, a phase 2 clinical trial (NCT00112580) showed that the intravenous administration of ipilimumab (3.0 mg/kg every 3 weeks; four doses/course for a maximum of two courses) was not effective for the treatment of locally advanced or metastatic pancreas cancer [91]. Some antitumor treatments, such as FOLFIRINOX and gemcitabine, can significantly increase the expression of CD8^+^ T cells and decrease the expression of immunosuppressor cells and their secreted immunosuppressive cytokine TGF-β1 in PDAC patients [92,93], including Treg cells. However, the depletion of Tregs shows contradictory results in murine PDAC models. For example, treatment with C-C motif chemokine receptor 5 (CCR5) inhibitor TAK-779 suppressed Treg migration to tumors and inhibit tumor development in a murine PDAC model [94]. In contrast, in another mouse model, Treg depletion induced via diphtheria toxin (DT) injection caused increased myeloid cell infiltration and restored immunosuppression, resulting in tumor progression [68]. A recent study also showed that a higher infiltration of CD3^+^CD8^−^FOXP3^+^ Tregs was positively associated with increased OS in PDAC patients [95]. Therefore, the function of different subtypes of Tregs remains to be studied.

An accumulating number of studies are investigating the roles of different molecules in regulating Treg function and depletion. Treatment with an anti-glucocorticoid-induced TNF receptor (GITR) monoclonal antibody (mAb) can inhibit Treg function and tumor infiltration by downregulating CCR5 expression, resulting in the suppression of subcutaneous pancreatic tumor growth in a murine model. In addition, anti-GITR treatment shows synergy with IFN-α-mediated therapy [96]. The infiltration of LAG-3-expressing T cells is positively associated with reduced disease-free survival in PDAC patients [97]. Treatment with anti-LAG-3 antibody alone or in combination with anti-41BB antibody suppressed tumor growth and increased the survival time of C57BL/6 mice with orthotopic pancreatic tumors by increasing T cells and decreasing immunosuppressive myeloid cells [98]. Anti-CD25 treatment alone or in combination with anti-TGF-β can significantly suppress the tumor infiltration ability of Tregs in PDAC tissues, and dual treatment can increase CD8^+^ T cell infiltration and suppress tumor growth in PDAC murine models [99]. A clinical trial (NCT03621982) evaluated the efficacy of ADCT-301, an antibody–drug conjugate comprising a human monoclonal antibody against CD25 conjugated to a potent pyrrolobenzodiazepine dimer (PBD) toxin, in patients with advanced solid tumors, including pancreatic cancer. However, the trial was terminated due to no sufficient immunomodulatory activity being tested at a planned dose/time.

Forkheadbox protein 3 (Foxp3) was initially identified as a key transcription factor for Tregs that is also expressed in PDAC tumor cells (also known as cancer-Foxp3) and can upregulate the expression of C-C motif chemokine ligand 5 (CCL5) to recruit Treg cells into the tumor microenvironment [100]. Together with Tregs, the upregulation of PD-L1 expression in tumor cells induced by cancer-Foxp3 in PDAC increases immune evasion and causes the immunosuppressive microenvironment [101]. Treatment with a murine PD-1-targeted IL-2 variant antibody complex (PD1-IL2v), which is composed of a high-affinity anti-PD-1 antibody fused to an IL-2 variant with abolished binding to CD25 (IL-2Rα), can increase the expansion of tumor-antigen-specific CD8^+^ T cells, inhibit the immunosuppressive function of Tregs, and improve the antitumor efficacy of radiation therapy [102].

#### 3.1.3. Myeloid-Derived Suppressor Cells (MDSCs)

MDSCs are a heterogeneous population of myeloid cells in immune suppressive TMEs which are commonly associated with a reduced efficacy of immunotherapy and therapeutic resistance [103,104]. The inhibition of the growth factors and recruiting factors derived from tumor cells or the TME can inhibit the population of MDSCs in PDAC and their functions. For example, gemcitabine (GEM)-resistant PDAC cells can express higher levels of PD-L1 and granulocyte–macrophage colony-stimulating factor (GM-CSF) compared to parental PDAC cells, which can cause the accumulation of MDSCs and an immunosuppressive TME [105]. In cell and animal tumor models, a combination therapy with p53-expressing telomerase-specific oncolytic adenovirus OBP-702 and PD-L1 blockade or PD-1 blockade can improve the antitumor efficacy of PD-L1/PD-1 blockade and inhibit the GM-CSF-induced accumulation of MDSCs and immunosuppressive TME [105,106]. Chemokines/chemokine receptors such as CXCL2/CXCR2 and CCL2/CCR2 facilitate the recruitment of myeloid neutrophils and macrophages to PDAC to induce an immunosuppressive TME [107]. Treatment with CXCR1/2 inhibitor SX-682 dramatically depleted the population of intratumoral CXCR2^+^ MDSCs and increased the infiltration of CD8^+^ and CD4^+^ T cells to suppress pancreatic cancer growth in a mouse iKRAS PDAC model, enhancing the survival time of tumor-bearing mice [98]. Dual treatment with CCR2 and CXCR2 inhibitors can significantly increase the suppression of tumor-infiltrating myeloid cells in PDAC compared with either strategy alone and enhance the chemotherapeutic efficacy of FOLFIRINOX (5-Fluorouracil, irinotecan, and oxaliplatin) [107].

Phase 1 or 2 clinical trials have been designed to evaluate the tolerability and efficacy of a CCR2/5 dual inhibitor, BMS-813160, together with nivolumab, gemcitabine, and nab-paclitaxel in patients with borderline resectable and locally advanced PDAC (NCT03496662) or with/without GVAX (a GM-CSF gene-transfected tumor cell vaccine) following chemotherapy and radiotherapy in patients with locally advanced PDAC (NCT03767582). Phase 1 clinical trial results showed that a combined treatment consisting of BMS-813160 with nivolumab and GVAX (NCT03767582) was safe and did not cause a delay in surgical resection after neoadjuvant treatment [108].

### 3.2. Targeting Immunosuppressive Molecules

#### 3.2.1. Immune Checkpoints

The expression of immune checkpoints (ICs) such as PD-L1 and V-domain Ig suppressor of T cell activation (VISTA) is significantly correlated with the OS of patients with PDAC [109]. The LAG-3 expression of T cells in PDAC is correlated with reduced disease-free survival (DFS). PD-1 expression in PDAC-infiltrating Tregs is correlated with tumor metastasis to lymph nodes [110]. Currently, anti-PD-1 antibody pembrolizumab is the only FDA-approved treatment for patients with advanced PDAC [111]. Several clinical trials (NCT02546531, NCT02331251) have been initiated to evaluate the synergistic effect of pembrolizumab with chemotherapies and target therapies such as gemcitabine and defactinib [112,113]. Another pilot phase 1 clinical trial (NCT02311361) showed that immune checkpoint blockade (anti-PD-L1 antibody durvalumab or plus anti-CTLA-4 antibody tremelimumab) in combination with stereotactic body radiotherapy had a modest benefit in patients with metastatic PDAC [114]. One clinical trial has been initiated to test the efficacy and safety of anti-PD1 antibody toripalimab in combination with nab-paclitaxel and gemcitabine as a first-line treatment for patients with unresectable PDAC [115].

#### 3.2.2. TGF-β Pathway

TGF-β signaling plays an important role in tumor progression and metastasis in PDAC, as well as resistance to PDAC therapy. The role of TGF-β is dependent on the downstream signaling pathway and context-dependent [116]. TGF-β can cause a more aggressive phenotype and more severe infiltration of TAMs and increase the expression of PD-L1 in PDAC through non-SMAD signaling pathways [117]. Anti-TGF-β treatment alone or in combination with anti-CD25 can significantly suppress the concentration of TGF-β in PDAC tissues. In addition, the dual treatment can inhibit the tumor volumes in PDAC murine models [99]. In the TCGA database, the expression of *Cd274*-encoding PD-L1 in TAMs was also associated with the expression of *Tgb1* and *Tgfbr1* in PDAC [116]. Synergistic treatment involving the TGF-β receptor I (TGFβRI) kinase inhibitor (vactosertib, or EW-7197) can increase the antitumor activity of gemcitabine against pancreatic cancer cells compared to gemcitabine treatment alone and inhibit TGF-β/Smad2 signaling pathway to decrease the production of extracellular matrix (ECM) proteins such as collagens, fibronectin, and alpha-smooth muscle actin (α-SMA) [118]. Another study showed that when applied together with gemcitabine, treatment with galunisertib, an orally administered TGF-β type 1 receptor (ALK5) serine/threonine kinase inhibitor, improved the OS of patients with unresectable pancreatic cancer [119]. In addition, clinical trials also show that TGFβRI inhibitors such as galunisertib (NCT01373164) plus other treatments such as gemcitabine can improve the survival of patients with pancreatic cancer [120]. A phase I clinical trial (NCT02734160) showed that the co-administration of galunisertib (150 mg twice daily) with anti-PD-L1 antibody durvalumab (1500 mg on day 1 every 4 weeks) was safe in patients with refractory metastatic pancreatic cancer, with a disease control rate of 25% being recorded [121].

Treatment involving HCW9218, a bifunctional TGF-β antagonist showing IL-15 immunostimulatory activity, can increase the antitumor activity of chemotherapy nab-paclitaxel and gemcitabine and prolong the survival of pancreatic tumor-bearing mice [122]. A phase 1/2 clinal trial (NCT05304936) is recruiting to study the dose of HCW9218 monotherapy in patients with advanced/metastatic pancreatic cancer. For patients with metastatic PDAC who are resistant to the first-line treatment of gemcitabine and nab-paclitaxel, using the TGF-β receptor and ALK4/ALK5 inhibitor vactosertib in combination with FOLFOX (a combined chemotherapy regimen consisting of folinic acid, fluorouracil, and oxaliplatin) could be an optional treatment (NCT03666832).

#### 3.2.3. IL-10

As shown by the above-discussed studies, immunosuppressive cells such as Tregs, TAMs, and MDSCs can secrete IL-10 in the TME of PDAC to inhibit effector T cell functions [123]. In addition, PDAC cells can secrete IL-10 to induce the expression of the scavenger receptor macrophage receptor with collagenous structure (MARCO) in myeloid cells to suppress effector T cell and NK cell function [124]. Furthermore, IL-10 can suppress inflammation by inhibiting NF-κB signaling to exert an antitumor function [125]. A phase 1 clinical trial (NCT02009449) showed that pegilodecakin, a pegylated recombinant human IL-10, in combination with FOLFOX (folinic acid, fluorouracil, and oxaliplatin) has promising efficacy, showing a 2-year OS rate of 24% (95% CI: 10–42%) without causing immune-related adverse events [126]. However, a phase 3 clinical trial (NCT02923921) examined the efficacy of pegilodecakin (PEG), a pegylated recombinant human IL-10, in combination with FOLFOX (folinic acid, fluorouracil, and oxaliplatin) in patients with metastatic PDAC after first-line gemcitabine-containing therapy, but this approach did not result in an improvement in cancer therapy [127]. In addition, the above-discussed treatments that suppress immunosuppressive cells can also suppress the expression of IL-10 to enhance antitumor immunity.

#### 3.2.4. Focal Adhesion Kinase (FAK)

FAK is a tyrosine kinase and can regulate cancer progression and metastasis via regulating several cellular functions [128]. FAK is hyperactivated in the immunosuppressive TME of PDAC [129]. FAK can regulate the expression of immune checkpoints and the efficacy of checkpoint immunotherapy in mouse models of PDAC. For example, programmed death ligand 2 (PD-L2) has been shown to be positively associated with tumor progression and poor prognosis in patients with PDAC. FAK can upregulate IL-6 expression in pancreatic tumor cells, which acts synergistically with IL-4 from Th2 cells to drive the upregulation of PD-L2 in TAMs, dendritic cells (DCs), and endothelial cells [130]. In addition, activating the FAK signaling pathway in PDAC cells by CAF-derived β1-integrins can promote their clonogenic growth [129]. Furthermore, FAK inhibitors or their combination with immune checkpoint inhibitors can inhibit extracellular matrix production and the cell migration of CAFs and display antitumor effects in PDAC models [131,132]. Treatment with the FAK inhibitor VS-4718 decreased collagen production by decreasing the number of SMA-expressing fibroblasts and inhibiting the TGF-β/SMAD signaling pathway in PDAC [133]. FAK inhibition induced by the selective FAK inhibitor VS-4718 significantly prevented tumor progression and led to 2-fold increase in survival rate among KPC mice with Kras and single p53 mutation, mimicking human PDAC [70].

Several ongoing clinical trials are evaluating the efficacy of the FAK inhibitor defactinib (VS-6063) in combination with radiotherapy (stereotactic body radiotherapy, NCT04331041), or immunotherapy (αPD-1 antibody pembrolizumab, NCT02758587, and NCT03727880) for pancreatic cancer treatment. One clinical trial (NCT02546531) showed that a triple-drug treatment consisting of defactinib, pembrolizumab, and gemcitabine was well tolerated and had a disease control rate (DCR) of 80% in 20 patients with refractory PDAC [113]. The median progression-free survival (PFS) and OS of these patients were 3.6 and 7.8 months, respectively. The small molecule GSK2256098 (GlaxoSmithKline) can inhibit PDAC cell growth by targeting FAK Y397 phosphorylation [134], and it is under clinical evaluation in combination with trametinib (a MEK1/2 inhibitor) treatment for advanced pancreatic cancer (NCT02428270). A preclinical study showed that another FAK inhibitor, AMP945, in combination with FOLFIRINOX, can significantly increase the survival of pancreatic cancer-bearing mice with patient-derived xenograft (PDX) models by increasing tumor cell apoptosis and reducing FAK Y397 phosphorylation [135]. A clinical trial (NCT05355298) is recruiting to evaluate the efficacy and safety of AMP945 treatment in combination with nab-paclitaxel and gemcitabine in patients with unresectable or metastatic pancreatic cancer.

Overall, PDAC is highly resistant to chemotherapy compared to other tumors [136], which also enhances the treatment difficulty of immunotherapies due to the highly immunosuppressive TME caused by the above-mentioned factors. An accumulating number of treatments are showing the potent effects needed to combat this barrier (Table 2). Specifically, combinational treatment strategies incorporating strategies such as surgery operation, immunotherapy, chemotherapy, radiotherapy, and others have been shown to improve the antitumor efficacy of PDAC treatments. However, more clinical trials are required to evaluate these therapies.

## 4. Conclusions

Targeting the stromal barrier is a critical approach to overcoming drug resistance in PDAC. While this may be challenging, there are several opportunities for the development of novel targeted therapy in future research. The highly proliferative cellular components that secrete dense fibrous components are an opportunity for therapeutic intervention. These cells may contain receptors or surface glycoproteins that could be docked to regulate their activities. Further, the development of targeted inhibitors against aberrant pathway molecules that exacerbate the desmoplastic reaction may help to deconvolute the stroma barrier or resuscitate antitumor immunity. Thus, future research should focus on the identification of TME-specific actionable targets and the development of efficacious therapy against such targets to overcome drug resistance in PDAC.

## Figures and Tables

**Table 2:** Examples of treatments targeting immunosuppressive tumor microenvironment in PDAC.

Class of Therapies	Targets	Treatments	Clinical Trials	Phase
Immune checkpoint inhibitors	CTLA-4	Ipilimumab, an anti-CTLA-4 (cytotoxic T-lymphocyte-associated protein 4) antibody.	NCT00112580	2
	PD-1	Anti-PD-1 antibody pembrolizumab in combination with macrophage activator BXCL701.	NCT05558982	2
	PD-L1	Anti-PD-L1 antibody Durvalumab with a tyrosine kinase inhibitor of colony-stimulating factor 1 receptor (CSF1R) (pexidartinib).	NCT02777710	1
	PD-L1	Anti-PD-L1 antibody durvalumab co-administration with galunisertib.	NCT02734160	1
Cytokines/cytokine receptors	IL-10	Pegilodecakin, a pegylated recombinant human IL-10, in combination with FOLFOX (folinic acid, fluorouracil, and oxaliplatin).	NCT02009449	1
	TGF-β+IL-15	HCW9218, a bifunctional TGF-β antagonist comprising IL-15 immunostimulatory activity.	NCT05304936	1/2
	TGF-β receptor	TGF-β receptor I kinase inhibitor galunisertib plus gemcitabine.	NCT01373164	1/2
		TGFβ receptor I kinase inhibitor galunisertib plus the anti-PD-L1 antibody durvalumab.	NCT02734160	1
Chemokines/ chemokine receptors	CCR2/CCR5	CCR2/CCR5 inhibitor BMS-813160 in combination with nivolumab and gemcitabine and nab-paclitaxel.	NCT03496662	1/2
	CCR2	CCR2 inhibitor PF-04136309 in combination with chemotherapy FOLFIRINOX (oxaliplatin and irinotecan plus leucovorin and fluorouracil).	NCT01413022	1
	CCR2	CCR2-specific antagonist CCX872-B plus FOLFIRINOX.	NCT02345408	1
	CCR2	CCR2 inhibitor PF-04136309 in combination with chemotherapy FOLFIRINOX (oxaliplatin and irinotecan plus leucovorin and fluorouracil).	NCT01413022	1
Co-stimulatory molecules	CD25	ADCT-301, a monoclonal antibody that binds to CD25 conjugated to a PBD dimer toxin, in combination with or without anti-PD-1 therapy (pembrolizamab).	NCT03621982	1
Kinase inhibitors	CSF1R	Pexidartinib, a tyrosine kinase inhibitor of colony-stimulating factor 1 receptor (CSF1R).	NCT02777710	1
	FAK	Focal adhesion kinase (FAK) inhibitor defactinib with pembrolizumab.	NCT03727880	2
	FAK	Defactinib with stereotactic body radiotherapy (SBRT).	NCT04331041	2

## References

[1] Ma S., Sokale I.O., Thrift A.P. (2023). Trends and Variations in Pancreatic Cancer Mortality Among US Metro and Nonmetro Adults, 1999–2020. J Clin Gastroenterol.

[2] Aier I., Semwal R., Sharma A., Varadwaj P.K. (2019). A systematic assessment of statistics, risk factors, and underlying features involved in pancreatic cancer. Cancer Epidemiol..

[3] Sherman M.H., Beatty G.L. (2023). Tumor Microenvironment in Pancreatic Cancer Pathogenesis and Therapeutic Resistance. Annu. Rev. Pathol..

[4] Hartupee C., Nagalo B.M., Chabu C.Y., Tesfay M.Z., Coleman-Barnett J., West J.T., Moaven O. (2024). Pancreatic cancer tumor microenvironment is a major therapeutic barrier and target. Front. Immunol..

[5] Ho W.J., Jaffee E.M., Zheng L. (2020). The tumour microenvironment in pancreatic cancer—Clinical challenges and opportunities. Nat. Rev. Clin. Oncol..

[6] Whatcott C.J., Posner R.G., Von Hoff D.D., Han H., Grippo P.J., Munshi H.G. (2012). Desmoplasia and chemoresistance in pancreatic cancer. Pancreatic Cancer and Tumor Microenvironment.

[7] Edwards P., Kang B.W., Chau I. (2021). Targeting the Stroma in the Management of Pancreatic Cancer. Front. Oncol..

[8] Sato N., Cheng X.B., Kohi S., Koga A., Hirata K. (2016). Targeting hyaluronan for the treatment of pancreatic ductal adenocarcinoma. Acta Pharm. Sin. B.

[9] Kim P.K., Halbrook C.J., Kerk S.A., Radyk M., Wisner S., Kremer D.M., Sajjakulnukit P., Andren A., Hou S.W., Trivedi A. (2021). Hyaluronic acid fuels pancreatic cancer cell growth. eLife.

[10] Cheng X.B., Sato N., Kohi S., Koga A., Hirata K. (2018). 4-Methylumbelliferone inhibits enhanced hyaluronan synthesis and cell migration in pancreatic cancer cells in response to tumor-stromal interactions. Oncol. Lett..

[11] Yoshida E., Kudo D., Nagase H., Suto A., Shimoda H., Suto S., Kakizaki I., Endo M., Hakamada K. (2018). 4-Methylumbelliferone Decreases the Hyaluronan-rich Extracellular Matrix and Increases the Effectiveness of 5-Fluorouracil. Anticancer Res..

[12] Jiang W., Zhang Y., Kane K.T., Collins M.A., Simeone D.M., Di Magliano M.P., Nguyen K.T. (2015). CD44 regulates pancreatic cancer invasion through MT1-MMP. Mol. Cancer Res..

[13] Menke-van der Houven van Oordt C.W., Gomez-Roca C., Van Herpen C., Coveler A.L., Mahalingam D., Verheul H.M., Van der Graaf W.T., Christen R., Rüttinger D., Weigand S. (2016). First-in-human phase I clinical trial of RG7356, an anti-CD44 humanized antibody, in patients with advanced, CD44-expressing solid tumors. Oncotarget.

[14] Gold M.A., Brady W.E., Lankes H.A., Rose P.G., Kelley J.L., De Geest K., Crispens M.A., Resnick K.E., Howell S.B. (2012). A phase II study of a urokinase-derived peptide (A6) in the treatment of persistent or recurrent epithelial ovarian, fallopian tube, or primary peritoneal carcinoma: A Gynecologic Oncology Group study. Gynecol. Oncol..

[15] Tijink B.M., Buter J., De Bree R., Giaccone G., Lang M.S., Staab A., Leemans C.R., Van Dongen G.A. (2006). A phase I dose escalation study with anti-CD44v6 bivatuzumab mertansine in patients with incurable squamous cell carcinoma of the head and neck or esophagus. Clin. Cancer Res..

[16] Gouda H.M., Abdel Mohsen M.M. (2009). Frequency of expression of RHAMM/CD168 in Egyptian patients with CML. J. Egypt. Natl. Canc Inst..

[17] Giannopoulos K., Własiuk P., Dmoszyńska A., Roliński J., Schmitt M. (2011). Peptide vaccination induces profound changes in the immune system in patients with B-cell chronic lymphocytic leukemia. Folia Histochem. Cytobiol..

[18] Shi Y., Reiman T., Li W., Maxwell C.A., Sen S., Pilarski L., Daniels T.R., Penichet M.L., Feldman R., Lichtenstein A. (2007). Targeting aurora kinases as therapy in multiple myeloma. Blood.

[19] Provenzano P.P., Cuevas C., Chang A.E., Goel V.K., Von Hoff D.D., Hingorani S.R. (2012). Enzymatic targeting of the stroma ablates physical barriers to treatment of pancreatic ductal adenocarcinoma. Cancer Cell.

[20] Heineman T., Baumgart M., Nanavati C., Gabrail N., Van Wart S.A., Mager D.E., Maneval D.C., Fathallah A.M., Sekulovich R.E. (2021). Safety and pharmacokinetics of docetaxel in combination with pegvorhyaluronidase alfa in patients with non-small cell lung cancer. Clin. Transl. Sci..

[21] Hingorani S.R., Harris W.P., Beck J.T., Berdov B.A., Wagner S.A., Pshevlotsky E.M., Tjulandin S.A., Gladkov O.A., Holcombe R.F., Korn R. (2016). Phase Ib Study of PEGylated Recombinant Human Hyaluronidase and Gemcitabine in Patients with Advanced Pancreatic Cancer. Clin. Cancer Res..

[22] Infante J.R., Korn R.L., Rosen L.S., LoRusso P., Dychter S.S., Zhu J., Maneval D.C., Jiang P., Shepard H.M., Frost G. (2018). Phase 1 trials of PEGylated recombinant human hyaluronidase PH20 in patients with advanced solid tumours. Br. J. Cancer.

[23] Ramanathan R.K., McDonough S.L., Philip P.A., Hingorani S.R., Lacy J., Kortmansky J.S., Thumar J., Chiorean E.G., Shields A.F., Behl D. (2019). Phase IB/II Randomized Study of FOLFIRINOX Plus Pegylated Recombinant Human Hyaluronidase Versus FOLFIRINOX Alone in Patients With Metastatic Pancreatic Adenocarcinoma: SWOG S1313. J. Clin. Oncol..

[24] Ko A.H., Kim K.P., Siveke J.T., Lopez C.D., Lacy J., O’Reilly E.M., Macarulla T., Manji G.A., Lee J., Ajani J. (2023). Atezolizumab Plus PEGPH20 Versus Chemotherapy in Advanced Pancreatic Ductal Adenocarcinoma and Gastric Cancer: MORPHEUS Phase Ib/II Umbrella Randomized Study Platform. Oncologist.

[25] Doherty G.J., Tempero M., Corrie P.G. (2018). HALO-109-301: A Phase III trial of PEGPH20 (with gemcitabine and nab-paclitaxel) in hyaluronic acid-high stage IV pancreatic cancer. Future Oncol..

[26] Van Cutsem E., Tempero M.A., Sigal D., Oh D.Y., Fazio N., Macarulla T., Hitre E., Hammel P., Hendifar A.E., Bates S.E. (2020). Randomized Phase III Trial of Pegvorhyaluronidase Alfa With Nab-Paclitaxel Plus Gemcitabine for Patients With Hyaluronan-High Metastatic Pancreatic Adenocarcinoma. J. Clin. Oncol..

[27] Hamidi H., Ivaska J. (2018). Every step of the way: Integrins in cancer progression and metastasis. Nat. Rev. Cancer.

[28] Dean A., Gill S., McGregor M., Broadbridge V., Järveläinen H.A., Price T. (2022). Dual αV-integrin and neuropilin-1 targeting peptide CEND-1 plus nab-paclitaxel and gemcitabine for the treatment of metastatic pancreatic ductal adenocarcinoma: A first-in-human, open-label, multicentre, phase 1 study. Lancet Gastroenterol. Hepatol..

[29] Keizer R.J., Funahashi Y., Semba T., Wanders J., Beijnen J.H., Schellens J.H., Huitema A.D. (2011). Evaluation of α2-integrin expression as a biomarker for tumor growth inhibition for the investigational integrin inhibitor E7820 in preclinical and clinical studies. Aaps J..

[30] Damhofer H., Medema J.P., Veenstra V.L., Badea L., Popescu I., Roelink H., Bijlsma M.F. (2013). Assessment of the stromal contribution to Sonic Hedgehog-dependent pancreatic adenocarcinoma. Mol. Oncol..

[31] Skoda A.M., Simovic D., Karin V., Kardum V., Vranic S., Serman L. (2018). The role of the Hedgehog signaling pathway in cancer: A comprehensive review. Bosn. J. Basic Med. Sci..

[32] Catenacci D.V., Junttila M.R., Karrison T., Bahary N., Horiba M.N., Nattam S.R., Marsh R., Wallace J., Kozloff M., Rajdev L. (2015). Randomized Phase Ib/II Study of Gemcitabine Plus Placebo or Vismodegib, a Hedgehog Pathway Inhibitor, in Patients with Metastatic Pancreatic Cancer. J. Clin. Oncol..

[33] Gajjar A., Stewart C.F., Ellison D.W., Kaste S., Kun L.E., Packer R.J., Goldman S., Chintagumpala M., Wallace D., Takebe N. (2013). Phase I study of vismodegib in children with recurrent or refractory medulloblastoma: A pediatric brain tumor consortium study. Clin. Cancer Res..

[34] Robinson G.W., Orr B.A., Wu G., Gururangan S., Lin T., Qaddoumi I., Packer R.J., Goldman S., Prados M.D., Desjardins A. (2015). Vismodegib Exerts Targeted Efficacy Against Recurrent Sonic Hedgehog-Subgroup Medulloblastoma: Results From Phase II Pediatric Brain Tumor Consortium Studies PBTC-025B and PBTC-032. J. Clin. Oncol..

[35] Kim E.J., Sahai V., Abel E.V., Griffith K.A., Greenson J.K., Takebe N., Khan G.N., Blau J.L., Craig R., Balis U.G. (2014). Pilot clinical trial of hedgehog pathway inhibitor GDC-0449 (vismodegib) in combination with gemcitabine in patients with metastatic pancreatic adenocarcinoma. Clin. Cancer Res..

[36] Frappaz D., Barritault M., Montané L., Laigle-Donadey F., Chinot O., Le Rhun E., Bonneville-Levard A., Hottinger A.F., Meyronnet D., Bidaux A.S. (2021). MEVITEM-a phase I/II trial of vismodegib + temozolomide vs temozolomide in patients with recurrent/refractory medulloblastoma with Sonic Hedgehog pathway activation. Neuro-Oncology.

[37] Ueno H., Kondo S., Yoshikawa S., Inoue K., Andre V., Tajimi M., Murakami H. (2018). A phase I and pharmacokinetic study of taladegib, a Smoothened inhibitor, in Japanese patients with advanced solid tumors. Investig. New Drugs.

[38] Kieran M.W., Chisholm J., Casanova M., Brandes A.A., Aerts I., Bouffet E., Bailey S., Leary S., MacDonald T.J., Mechinaud F. (2017). Phase I study of oral sonidegib (LDE225) in pediatric brain and solid tumors and a phase II study in children and adults with relapsed medulloblastoma. Neuro-Oncology.

[39] Liu F., Lagares D., Choi K.M., Stopfer L., Marinković A., Vrbanac V., Probst C.K., Hiemer S.E., Sisson T.H., Horowitz J.C. (2015). Mechanosignaling through YAP and TAZ drives fibroblast activation and fibrosis. Am. J. Physiol. Lung Cell Mol. Physiol..

[40] Ansari D., Ohlsson H., Althini C., Bauden M., Zhou Q., Hu D., Andersson R. (2019). The Hippo Signaling Pathway in Pancreatic Cancer. Anticancer Res..

[41] Higashi T., Hayashi H., Kitano Y., Yamamura K., Kaida T., Arima K., Taki K., Nakagawa S., Okabe H., Nitta H. (2016). Statin attenuates cell proliferative ability via TAZ (WWTR1) in hepatocellular carcinoma. Med. Oncol..

[42] Hanahan D., Weinberg R.A. (2011). Hallmarks of cancer: The next generation. Cell.

[43] Boyd L.N.C., Andini K.D., Peters G.J., Kazemier G., Giovannetti E. (2022). Heterogeneity and plasticity of cancer-associated fibroblasts in the pancreatic tumor microenvironment. Semin. Cancer Biol..

[44] Özdemir B.C., Pentcheva-Hoang T., Carstens J.L., Zheng X., Wu C.C., Simpson T.R., Laklai H., Sugimoto H., Kahlert C., Novitskiy S.V. (2014). Depletion of carcinoma-associated fibroblasts and fibrosis induces immunosuppression and accelerates pancreas cancer with reduced survival. Cancer Cell.

[45] Tran E., Chinnasamy D., Yu Z., Morgan R.A., Lee C.C., Restifo N.P., Rosenberg S.A. (2013). Immune targeting of fibroblast activation protein triggers recognition of multipotent bone marrow stromal cells and cachexia. J. Exp. Med..

[46] McAndrews K.M., Chen Y., Darpolor J.K., Zheng X., Yang S., Carstens J.L., Li B., Wang H., Miyake T., Correa de Sampaio P. (2022). Identification of Functional Heterogeneity of Carcinoma-Associated Fibroblasts with Distinct IL6-Mediated Therapy Resistance in Pancreatic Cancer. Cancer Discov..

[47] Heichler C., Scheibe K., Schmied A., Geppert C.I., Schmid B., Wirtz S., Thoma O.M., Kramer V., Waldner M.J., Büttner C. (2020). STAT3 activation through IL-6/IL-11 in cancer-associated fibroblasts promotes colorectal tumour development and correlates with poor prognosis. Gut.

[48] Hurwitz H., Van Cutsem E., Bendell J., Hidalgo M., Li C.P., Salvo M.G., Macarulla T., Sahai V., Sama A., Greeno E. (2018). Ruxolitinib + capecitabine in advanced/metastatic pancreatic cancer after disease progression/intolerance to first-line therapy: JANUS 1 and 2 randomized phase III studies. Investig. New Drugs.

[49] Zhuang Z., Ju H.Q., Aguilar M., Gocho T., Li H., Iida T., Lee H., Fan X., Zhou H., Ling J. (2016). IL1 Receptor Antagonist Inhibits Pancreatic Cancer Growth by Abrogating NF-κB Activation. Clin. Cancer Res..

[50] Hu C., Xia R., Zhang X., Li T., Ye Y., Li G., He R., Li Z., Lin Q., Zheng S. (2022). circFARP1 enables cancer-associated fibroblasts to promote gemcitabine resistance in pancreatic cancer via the LIF/STAT3 axis. Mol. Cancer.

[51] Kang X., Lin Z., Xu M., Pan J., Wang Z.W. (2019). Deciphering role of FGFR signalling pathway in pancreatic cancer. Cell Prolif..

[52] Meric-Bernstam F., Bahleda R., Hierro C., Sanson M., Bridgewater J., Arkenau H.T., Tran B., Kelley R.K., Park J.O., Javle M. (2022). Futibatinib, an Irreversible FGFR1-4 Inhibitor, in Patients with Advanced Solid Tumors Harboring FGF/FGFR Aberrations: A Phase I Dose-Expansion Study. Cancer Discov..

[53] Subbiah V., Iannotti N.O., Gutierrez M., Smith D.C., Féliz L., Lihou C.F., Tian C., Silverman I.M., Ji T., Saleh M. (2022). FIGHT-101, a first-in-human study of potent and selective FGFR 1-3 inhibitor pemigatinib in pan-cancer patients with FGF/FGFR alterations and advanced malignancies. Ann. Oncol..

[54] Ma W.W., Xie H., Fetterly G., Pitzonka L., Whitworth A., LeVea C., Wilton J., Mantione K., Schihl S., Dy G.K. (2019). A Phase Ib Study of the FGFR/VEGFR Inhibitor Dovitinib With Gemcitabine and Capecitabine in Advanced Solid Tumor and Pancreatic Cancer Patients. Am. J. Clin. Oncol..

[55] Ueno M., Ikeda M., Sasaki T., Nagashima F., Mizuno N., Shimizu S., Ikezawa H., Hayata N., Nakajima R., Morizane C. (2020). Phase 2 study of lenvatinib monotherapy as second-line treatment in unresectable biliary tract cancer: Primary analysis results. BMC Cancer.

[56] Izumi D., Ishimoto T., Miyake K., Sugihara H., Eto K., Sawayama H., Yasuda T., Kiyozumi Y., Kaida T., Kurashige J. (2016). CXCL12/CXCR4 activation by cancer-associated fibroblasts promotes integrin β1 clustering and invasiveness in gastric cancer. Int. J. Cancer.

[57] Fearon D.T., Janowitz T. (2021). AMD3100/Plerixafor overcomes immune inhibition by the CXCL12-KRT19 coating on pancreatic and colorectal cancer cells. Br. J. Cancer.

[58] Bockorny B., Semenisty V., Macarulla T., Borazanci E., Wolpin B.M., Stemmer S.M., Golan T., Geva R., Borad M.J., Pedersen K.S. (2020). BL-8040, a CXCR4 antagonist, in combination with pembrolizumab and chemotherapy for pancreatic cancer: The COMBAT trial. Nat. Med..

[59] Diop-Frimpong B., Chauhan V.P., Krane S., Boucher Y., Jain R.K. (2011). Losartan inhibits collagen I synthesis and improves the distribution and efficacy of nanotherapeutics in tumors. Proc. Natl. Acad. Sci. USA.

[60] Takabatake K., Shimo T., Murakami J., Anqi C., Kawai H., Yoshida S., Wathone Oo M., Haruka O., Sukegawa S., Tsujigiwa H. (2019). The Role of Sonic Hedgehog Signaling in the Tumor Microenvironment of Oral Squamous Cell Carcinoma. Int. J. Mol. Sci..

[61] Cazet A.S., Hui M.N., Elsworth B.L., Wu S.Z., Roden D., Chan C.L., Skhinas J.N., Collot R., Yang J., Harvey K. (2018). Targeting stromal remodeling and cancer stem cell plasticity overcomes chemoresistance in triple negative breast cancer. Nat. Commun..

[62] Sharma M., Turaga R.C., Yuan Y., Satyanarayana G., Mishra F., Bian Z., Liu W., Sun L., Yang J., Liu Z.R. (2021). Simultaneously targeting cancer-associated fibroblasts and angiogenic vessel as a treatment for TNBC. J. Exp. Med..

[63] Huang T.X., Tan X.Y., Huang H.S., Li Y.T., Liu B.L., Liu K.S., Chen X., Chen Z., Guan X.Y., Zou C. (2022). Targeting cancer-associated fibroblast-secreted WNT2 restores dendritic cell-mediated antitumour immunity. Gut.

[64] Munshi N.C., Anderson L.D., Shah N., Madduri D., Berdeja J., Lonial S., Raje N., Lin Y., Siegel D., Oriol A. (2021). Idecabtagene Vicleucel in Relapsed and Refractory Multiple Myeloma. N. Engl. J. Med..

[65] Sakemura R., Hefazi M., Siegler E.L., Cox M.J., Larson D.P., Hansen M.J., Manriquez Roman C., Schick K.J., Can I., Tapper E.E. (2022). Targeting cancer-associated fibroblasts in the bone marrow prevents resistance to CART-cell therapy in multiple myeloma. Blood.

[66] Horwitz S.M., Koch R., Porcu P., Oki Y., Moskowitz A., Perez M., Myskowski P., Officer A., Jaffe J.D., Morrow S.N. (2018). Activity of the PI3K-δ,γ inhibitor duvelisib in a phase 1 trial and preclinical models of T-cell lymphoma. Blood.

[67] Karamitopoulou E. (2019). Tumour microenvironment of pancreatic cancer: Immune landscape is dictated by molecular and histopathological features. Br. J. Cancer.

[68] Zhang Y., Lazarus J., Steele N.G., Yan W., Lee H.J., Nwosu Z.C., Halbrook C.J., Menjivar R.E., Kemp S.B., Sirihorachai V.R. (2020). Regulatory T-cell Depletion Alters the Tumor Microenvironment and Accelerates Pancreatic Carcinogenesis. Cancer Discov..

[69] Shibuya K.C., Goel V.K., Xiong W., Sham J.G., Pollack S.M., Leahy A.M., Whiting S.H., Yeh M.M., Yee C., Riddell S.R. (2014). Pancreatic ductal adenocarcinoma contains an effector and regulatory immune cell infiltrate that is altered by multimodal neoadjuvant treatment. PLoS ONE.

[70] Jiang H., Hegde S., Knolhoff B.L., Zhu Y., Herndon J.M., Meyer M.A., Nywening T.M., Hawkins W.G., Shapiro I.M., Weaver D.T. (2016). Targeting focal adhesion kinase renders pancreatic cancers responsive to checkpoint immunotherapy. Nat. Med..

[71] Yang S., Liu Q., Liao Q. (2020). Tumor-Associated Macrophages in Pancreatic Ductal Adenocarcinoma: Origin, Polarization, Function, and Reprogramming. Front. Cell Dev. Biol..

[72] Biffi G., Oni T.E., Spielman B., Hao Y., Elyada E., Park Y., Preall J., Tuveson D.A. (2019). IL1-Induced JAK/STAT Signaling Is Antagonized by TGFβ to Shape CAF Heterogeneity in Pancreatic Ductal Adenocarcinoma. Cancer Discov..

[73] Zhang C., Yang M., Ericsson A.C. (2021). Function of Macrophages in Disease: Current Understanding on Molecular Mechanisms. Front. Immunol..

[74] Xia Q., Jia J., Hu C., Lu J., Li J., Xu H., Fang J., Feng D., Wang L., Chen Y. (2022). Tumor-associated macrophages promote PD-L1 expression in tumor cells by regulating PKM2 nuclear translocation in pancreatic ductal adenocarcinoma. Oncogene.

[75] Alausa A., Lawal K.A., Babatunde O.A., Obiwulu E.N.O., Oladokun O.C., Fadahunsi O.S., Celestine U.O., Moses E.U., Akaniro I.R., Adegbola P.I. (2022). Overcoming immunotherapeutic resistance in PDAC: SIRPα-CD47 blockade. Pharmacol. Res..

[76] Xi Q., Zhang J., Yang G., Zhang L., Chen Y., Wang C., Zhang Z., Guo X., Zhao J., Xue Z. (2020). Restoration of miR-340 controls pancreatic cancer cell CD47 expression to promote macrophage phagocytosis and enhance antitumor immunity. J. Immunother. Cancer.

[77] Barkal A.A., Brewer R.E., Markovic M., Kowarsky M., Barkal S.A., Zaro B.W., Krishnan V., Hatakeyama J., Dorigo O., Barkal L.J. (2019). CD24 signalling through macrophage Siglec-10 is a target for cancer immunotherapy. Nature.

[78] Gu H., Deng W., Zhang Y., Chang Y., Shelat V.G., Tsuchida K., Lino-Silva L.S., Wang Z. (2022). NLRP3 activation in tumor-associated macrophages enhances lung metastasis of pancreatic ductal adenocarcinoma. Transl. Lung Cancer Res..

[79] Karnell J.L., Rieder S.A., Ettinger R., Kolbeck R. (2019). Targeting the CD40-CD40L pathway in autoimmune diseases: Humoral immunity and beyond. Adv. Drug Deliv. Rev..

[80] Nguyen V.T., Benveniste E.N. (2000). Involvement of STAT-1 and ets family members in interferon-gamma induction of CD40 transcription in microglia/macrophages. J. Biol. Chem..

[81] Soong R.S., Song L., Trieu J., Lee S.Y., He L., Tsai Y.C., Wu T.C., Hung C.F. (2014). Direct T cell activation via CD40 ligand generates high avidity CD8+ T cells capable of breaking immunological tolerance for the control of tumors. PLoS ONE.

[82] Van Essen D., Kikutani H., Gray D. (1995). CD40 ligand-transduced co-stimulation of T cells in the development of helper function. Nature.

[83] Beatty G.L., Chiorean E.G., Fishman M.P., Saboury B., Teitelbaum U.R., Sun W., Huhn R.D., Song W., Li D., Sharp L.L. (2011). CD40 agonists alter tumor stroma and show efficacy against pancreatic carcinoma in mice and humans. Science.

[84] Morrison A.H., Diamond M.S., Hay C.A., Byrne K.T., Vonderheide R.H. (2020). Sufficiency of CD40 activation and immune checkpoint blockade for T cell priming and tumor immunity. Proc. Natl. Acad. Sci. USA.

[85] Byrne K.T., Betts C.B., Mick R., Sivagnanam S., Bajor D.L., Laheru D.A., Chiorean E.G., O’Hara M.H., Liudahl S.M., Newcomb C. (2021). Neoadjuvant Selicrelumab, an Agonist CD40 Antibody, Induces Changes in the Tumor Microenvironment in Patients with Resectable Pancreatic Cancer. Clin. Cancer Res..

[86] Nywening T.M., Wang-Gillam A., Sanford D.E., Belt B.A., Panni R.Z., Cusworth B.M., Toriola A.T., Nieman R.K., Worley L.A., Yano M. (2016). Targeting tumour-associated macrophages with CCR2 inhibition in combination with FOLFIRINOX in patients with borderline resectable and locally advanced pancreatic cancer: A single-centre, open-label, dose-finding, non-randomised, phase 1b trial. Lancet Oncol..

[87] Linehan D., Noel M.S., Hezel A.F., Wang-Gillam A., Eskens F., Sleijfer S., Desar I.M.E., Erdkamp F., Wilmink J., Diehl J. (2018). Overall survival in a trial of orally administered CCR2 inhibitor CCX872 in locally advanced/metastatic pancreatic cancer: Correlation with blood monocyte counts. J. Clin. Oncol..

[88] Cassier P.A., Garin G., Eberst L., Delord J.-P., Chabaud S., Terret C., Montane L., Bidaux A.-S., Laurent S., Jaubert L. (2019). MEDIPLEX: A phase 1 study of durvalumab (D) combined with pexidartinib (P) in patients (pts) with advanced pancreatic ductal adenocarcinoma (PDAC) and colorectal cancer (CRC). J. Clin. Oncol..

[89] Gao Z., Zhang Q., Zhang X., Song Y. (2022). Advance of T regulatory cells in tumor microenvironment remodeling and immunotherapy in pancreatic cancer. Eur. J. Inflamm..

[90] Yano H., Thakur A., Tomaszewski E.N., Choi M., Deol A., Lum L.G. (2014). Ipilimumab augments antitumor activity of bispecific antibody-armed T cells. J. Transl. Med..

[91] Royal R.E., Levy C., Turner K., Mathur A., Hughes M., Kammula U.S., Sherry R.M., Topalian S.L., Yang J.C., Lowy I. (2010). Phase 2 trial of single agent Ipilimumab (anti-CTLA-4) for locally advanced or metastatic pancreatic adenocarcinoma. J. Immunother..

[92] Peng H., James C.A., Cullinan D.R., Hogg G.D., Mudd J.L., Zuo C., Takchi R., Caldwell K.E., Liu J., DeNardo D.G. (2021). Neoadjuvant FOLFIRINOX Therapy Is Associated with Increased Effector T Cells and Reduced Suppressor Cells in Patients with Pancreatic Cancer. Clin. Cancer Res..

[93] Eriksson E., Wenthe J., Irenaeus S., Loskog A., Ullenhag G. (2016). Gemcitabine reduces MDSCs, tregs and TGFβ-1 while restoring the teff/treg ratio in patients with pancreatic cancer. J. Transl. Med..

[94] Tan M.C., Goedegebuure P.S., Belt B.A., Flaherty B., Sankpal N., Gillanders W.E., Eberlein T.J., Hsieh C.S., Linehan D.C. (2009). Disruption of CCR5-dependent homing of regulatory T cells inhibits tumor growth in a murine model of pancreatic cancer. J. Immunol..

[95] Brouwer T., Ijsselsteijn M., Oosting J., Ruano D., Van der Ploeg M., Dijk F., Bonsing B., Fariña A., Morreau H., Vahrmeijer A. (2022). A Paradoxical Role for Regulatory T Cells in the Tumor Microenvironment of Pancreatic Cancer. Cancers.

[96] Aida K., Miyakawa R., Suzuki K., Narumi K., Udagawa T., Yamamoto Y., Chikaraishi T., Yoshida T., Aoki K. (2014). Suppression of Tregs by anti-glucocorticoid induced TNF receptor antibody enhances the antitumor immunity of interferon-α gene therapy for pancreatic cancer. Cancer Sci..

[97] Seifert L., Plesca I., Müller L., Sommer U., Heiduk M., Von Renesse J., Digomann D., Glück J., Klimova A., Weitz J. (2021). LAG-3-Expressing Tumor-Infiltrating T Cells Are Associated with Reduced Disease-Free Survival in Pancreatic Cancer. Cancers.

[98] Gulhati P., Schalck A., Jiang S., Shang X., Wu C.-J., Hou P., Ruiz S.H., Soto L.S., Parra E., Ying H. (2023). Targeting T cell checkpoints 41BB and LAG3 and myeloid cell CXCR1/CXCR2 results in antitumor immunity and durable response in pancreatic cancer. Nat. Cancer.

[99] Pu N., Zhao G., Yin H., Li J.A., Nuerxiati A., Wang D., Xu X., Kuang T., Jin D., Lou W. (2018). CD25 and TGF-β blockade based on predictive integrated immune ratio inhibits tumor growth in pancreatic cancer. J. Transl. Med..

[100] Wang X., Lang M., Zhao T., Feng X., Zheng C., Huang C., Hao J., Dong J., Luo L., Li X. (2017). Cancer-FOXP3 directly activated CCL5 to recruit FOXP3+Treg cells in pancreatic ductal adenocarcinoma. Oncogene.

[101] Wang X., Li X., Wei X., Jiang H., Lan C., Yang S., Wang H., Yang Y., Tian C., Xu Z. (2020). PD-L1 is a direct target of cancer-FOXP3 in pancreatic ductal adenocarcinoma (PDAC), and combined immunotherapy with antibodies against PD-L1 and CCL5 is effective in the treatment of PDAC. Signal Transduct. Target. Ther..

[102] Piper M., Hoen M., Darragh L.B., Knitz M.W., Nguyen D., Gadwa J., Durini G., Karakoc I., Grier A., Neupert B. (2023). Simultaneous targeting of PD-1 and IL-2Rβγ with radiation therapy inhibits pancreatic cancer growth and metastasis. Cancer Cell.

[103] Porembka M.R., Mitchem J.B., Belt B.A., Hsieh C.S., Lee H.M., Herndon J., Gillanders W.E., Linehan D.C., Goedegebuure P. (2012). Pancreatic adenocarcinoma induces bone marrow mobilization of myeloid-derived suppressor cells which promote primary tumor growth. Cancer Immunol. Immunother..

[104] Banerjee K., Kumar S., Ross K.A., Gautam S., Poelaert B., Nasser M.W., Aithal A., Bhatia R., Wannemuehler M.J., Narasimhan B. (2018). Emerging trends in the immunotherapy of pancreatic cancer. Cancer Lett..

[105] Kajiwara Y., Tazawa H., Yamada M., Kanaya N., Fushimi T., Kikuchi S., Kuroda S., Ohara T., Noma K., Yoshida R. (2023). Oncolytic virus-mediated reducing of myeloid-derived suppressor cells enhances the efficacy of PD-L1 blockade in gemcitabine-resistant pancreatic cancer. Cancer Immunol. Immunother..

[106] Araki H., Tazawa H., Kanaya N., Kajiwara Y., Yamada M., Hashimoto M., Kikuchi S., Kuroda S., Yoshida R., Umeda Y. (2022). Oncolytic virus-mediated p53 overexpression promotes immunogenic cell death and efficacy of PD-1 blockade in pancreatic cancer. Mol. Ther. Oncolytics.

[107] Nywening T.M., Belt B.A., Cullinan D.R., Panni R.Z., Han B.J., Sanford D.E., Jacobs R.C., Ye J., Patel A.A., Gillanders W.E. (2018). Targeting both tumour-associated CXCR2(+) neutrophils and CCR2(+) macrophages disrupts myeloid recruitment and improves chemotherapeutic responses in pancreatic ductal adenocarcinoma. Gut.

[108] Christenson E., Lim S.J., Wang H., Ferguson A., Parkinson R., Cetasaan Y., Rodriguez C., Burkhart R., De Jesus-Acosta A., He J. (2023). Nivolumab and a CCR2/CCR5 dual antagonist (BMS-813160) with or without GVAX for locally advanced pancreatic ductal adenocarcinomas: Results of phase I study. J. Clin. Oncol..

[109] Loch F.N., Kamphues C., Beyer K., Schineis C., Rayya W., Lauscher J.C., Horst D., Dragomir M.P., Schallenberg S. (2023). The Immune Checkpoint Landscape in Tumor Cells of Pancreatic Ductal Adenocarcinoma. Int. J. Mol. Sci..

[110] Seifert A.M., Eymer A., Heiduk M., Wehner R., Tunger A., Von Renesse J., Decker R., Aust D.E., Welsch T., Reissfelder C. (2020). PD-1 Expression by Lymph Node and Intratumoral Regulatory T Cells Is Associated with Lymph Node Metastasis in Pancreatic Cancer. Cancers.

[111] Bian J., Almhanna K. (2021). Pancreatic cancer and immune checkpoint inhibitors-still a long way to go. Transl. Gastroenterol. Hepatol..

[112] Weiss G.J., Blaydorn L., Beck J., Bornemann-Kolatzki K., Urnovitz H., Schütz E., Khemka V. (2018). Phase Ib/II study of gemcitabine, nab-paclitaxel, and pembrolizumab in metastatic pancreatic adenocarcinoma. Investig. New Drugs.

[113] Wang-Gillam A., Lim K.H., McWilliams R., Suresh R., Lockhart A.C., Brown A., Breden M., Belle J.I., Herndon J., Bogner S.J. (2022). Defactinib, Pembrolizumab, and Gemcitabine in Patients with Advanced Treatment Refractory Pancreatic Cancer: A Phase I Dose Escalation and Expansion Study. Clin. Cancer Res..

[114] Xie C., Duffy A.G., Brar G., Fioravanti S., Mabry-Hrones D., Walker M., Bonilla C.M., Wood B.J., Citrin D.E., Gil Ramirez E.M. (2020). Immune Checkpoint Blockade in Combination with Stereotactic Body Radiotherapy in Patients with Metastatic Pancreatic Ductal Adenocarcinoma. Clin. Cancer Res..

[115] Shui L., Cheng K., Li X., Shui P., Zhou X., Li J., Yi C., Cao D. (2020). Study protocol for an open-label, single-arm, phase Ib/II study of combination of toripalimab, nab-paclitaxel, and gemcitabine as the first-line treatment for patients with unresectable pancreatic ductal adenocarcinoma. BMC Cancer.

[116] Trebska-McGowan K., Chaib M., Alvarez M.A., Kansal R., Pingili A.K., Shibata D., Makowski L., Glazer E.S. (2022). TGF-β Alters the Proportion of Infiltrating Immune Cells in a Pancreatic Ductal Adenocarcinoma. J. Gastrointest. Surg..

[117] Hussain S.M., Kansal R.G., Alvarez M.A., Hollingsworth T.J., Elahi A., Miranda-Carboni G., Hendrick L.E., Pingili A.K., Albritton L.M., Dickson P.V. (2021). Role of TGF-β in pancreatic ductal adenocarcinoma progression and PD-L1 expression. Cell Oncol..

[118] Lee J.E., Lee P., Yoon Y.C., Han B.S., Ko S., Park M.S., Lee Y.J., Kim S.E., Cho Y.J., Lim J.H. (2023). Vactosertib, TGF-β receptor I inhibitor, augments the sensitization of the anti-cancer activity of gemcitabine in pancreatic cancer. Biomed. Pharmacother..

[119] Melisi D., Garcia-Carbonero R., Macarulla T., Pezet D., Deplanque G., Fuchs M., Trojan J., Oettle H., Kozloff M., Cleverly A. (2018). Galunisertib plus gemcitabine vs. gemcitabine for first-line treatment of patients with unresectable pancreatic cancer. Br. J. Cancer.

[120] Melisi D., Garcia-Carbonero R., Macarulla T., Pezet D., Deplanque G., Fuchs M., Trojan J., Kozloff M., Simionato F., Cleverly A. (2019). TGFβ receptor inhibitor galunisertib is linked to inflammation- and remodeling-related proteins in patients with pancreatic cancer. Cancer Chemother. Pharmacol..

[121] Melisi D., Oh D.Y., Hollebecque A., Calvo E., Varghese A., Borazanci E., Macarulla T., Merz V., Zecchetto C., Zhao Y. (2021). Safety and activity of the TGFβ receptor I kinase inhibitor galunisertib plus the anti-PD-L1 antibody durvalumab in metastatic pancreatic cancer. J. Immunother. Cancer.

[122] Chaturvedi P., George V., Shrestha N., Wang M., Dee M.J., Zhu X., Liu B., Egan J., D’Eramo F., Spanoudis C. (2022). Immunotherapeutic HCW9218 augments anti-tumor activity of chemotherapy via NK cell-mediated reduction of therapy-induced senescent cells. Mol. Ther..

[123] Li H.-B., Yang Z.-H., Guo Q.-Q. (2021). Immune checkpoint inhibition for pancreatic ductal adenocarcinoma: Limitations and prospects: A systematic review. Cell Commun. Signal..

[124] Sarhan D., Eisinger S., He F., Bergsland M., Pelicano C., Driescher C., Westberg K., Benitez I.I., Humoud R., Palano G. (2022). Targeting myeloid suppressive cells revives cytotoxic anti-tumor responses in pancreatic cancer. iScience.

[125] Padoan A., Plebani M., Basso D. (2019). Inflammation and Pancreatic Cancer: Focus on Metabolism, Cytokines, and Immunity. Int. J. Mol. Sci..

[126] Hecht J.R., Papadopoulos K.P., Falchook G.S., Patel M.R., Infante J.R., Aljumaily R., Wong D.J., Autio K.A., Wainberg Z.A., Bauer T.M. (2021). Immunologic and tumor responses of pegilodecakin with 5-FU/LV and oxaliplatin (FOLFOX) in pancreatic ductal adenocarcinoma (PDAC). Invest. New Drugs.

[127] Hecht J.R., Lonardi S., Bendell J., Sim H.W., Macarulla T., Lopez C.D., Van Cutsem E., Muñoz Martin A.J., Park J.O., Greil R. (2021). Randomized Phase III Study of FOLFOX Alone or With Pegilodecakin as Second-Line Therapy in Patients With Metastatic Pancreatic Cancer That Progressed After Gemcitabine (SEQUOIA). J. Clin. Oncol..

[128] Sulzmaier F.J., Jean C., Schlaepfer D.D. (2014). FAK in cancer: Mechanistic findings and clinical applications. Nat. Rev. Cancer.

[129] Begum A., McMillan R.H., Chang Y.T., Penchev V.R., Rajeshkumar N.V., Maitra A., Goggins M.G., Eshelman J.R., Wolfgang C.L., Rasheed Z.A. (2019). Direct Interactions With Cancer-Associated Fibroblasts Lead to Enhanced Pancreatic Cancer Stem Cell Function. Pancreas.

[130] Davidson C., Taggart D., Sims A.H., Lonergan D.W., Canel M., Serrels A. (2022). FAK promotes stromal PD-L2 expression associated with poor survival in pancreatic cancer. Br. J. Cancer.

[131] Zaghdoudi S., Decaup E., Belhabib I., Samain R., Cassant-Sourdy S., Rochotte J., Brunel A., Schlaepfer D., Cros J., Neuzillet C. (2020). FAK activity in cancer-associated fibroblasts is a prognostic marker and a druggable key metastatic player in pancreatic cancer. EMBO Mol. Med..

[132] Zhang C.Y., Liu S., Yang M. (2022). Clinical diagnosis and management of pancreatic cancer: Markers, molecular mechanisms, and treatment options. World J. Gastroenterol..

[133] Jiang H., Liu X., Knolhoff B.L., Hegde S., Lee K.B., Jiang H., Fields R.C., Pachter J.A., Lim K.H., DeNardo D.G. (2020). Development of resistance to FAK inhibition in pancreatic cancer is linked to stromal depletion. Gut.

[134] Zhang J., He D.H., Zajac-Kaye M., Hochwald S.N. (2014). A small molecule FAK kinase inhibitor, GSK2256098, inhibits growth and survival of pancreatic ductal adenocarcinoma cells. Cell Cycle.

[135] Burns C., Murphy K., Cock T.-A., Devlin M., Herrmann D., Timpson P. (2023). The effect of adding a selective FAK inhibitor AMP945 to FOLFIRINOX in a model of pancreatic cancer. J. Clin. Oncol..

[136] Springfeld C., Ferrone C.R., Katz M.H.G., Philip P.A., Hong T.S., Hackert T., Büchler M.W., Neoptolemos J. (2023). Neoadjuvant therapy for pancreatic cancer. Nat. Rev. Clin. Oncol..

